# Effects of 1,25-dihydroxyvitamin D_3_ on experimental periodontitis and AhR/NF-κB/NLRP3 inflammasome pathway in a mouse model

**DOI:** 10.1590/1678-7757-2018-0713

**Published:** 2019-11-04

**Authors:** Hao Li, Xinghua Zhong, Wei Li, Qi Wang

**Affiliations:** 1 Guangxi Medical University, the Affiliated Hospital of Stomatology, Department of Prosthodontics, China; 2 Sichuan University, West China Hospital of Stomatology, State Key Laboratory of Oral Diseases, China

**Keywords:** Periodontitis, Gingival epithelium, 1,25-dihydroxyvitamin D_3_, Aryl hydrocarbon receptor, NLR pyrin domain-containing 3 inflammasome

## Abstract

Vitamin D has been known to have important regulatory functions in inflammation and immune response and shows inhibitory effects on experimental periodontitis in animal models. However, the potential mechanism has yet to be clarified. Recent studies have highlighted Aryl hydrocarbon receptor (AhR) and its downstream signaling as a crucial regulator of immune homeostasis and inflammatory regulation. Objective: This study aimed to clarify the effect of 1,25-dihydroxyvitamin D_3_ (VD3) on experimental periodontitis and AhR/nuclear factor-κB (NF-κB)/NLR pyrin domain-containing 3 (NLRP3) inflammasome pathway in the gingival epithelium in a murine model. Methodology: We induced periodontitis in male C57BL/6 wild-type mice by oral inoculation of *Porphyromonas gingivalis* (*P. gingivalis*), and subsequently gave intraperitoneal VD3 injection to the mice every other day for 8 weeks. Afterwards, we examined the alveolar bone using scanning electron microscopy (SEM) and detected the gingival epithelial protein using western blot analysis and immunohistochemical staining. Results: SEM images demonstrated that alveolar bone loss was reduced in the periodontitis mouse model after VD3 supplementation. Western blot analyses and immunohistochemical staining of the gingival epithelium showed that the expression of vitamin D receptor, AhR and its downstream cytochrome P450 1A1 were enhanced upon VD3 application. Additionally, VD3 decreased NF-κB p65 phosphorylation, and NLRP3, apoptosis-associated speck-like protein, caspase-1, interleukin-1β (IL-1β) and IL-6 protein expression. Conclusions: These results implicate the alleviation of periodontitis and the alteration of AhR/NF-κB/NLRP3 inflammasome pathway by VD3 in the mouse model. The attenuation of this periodontal disease may correlate with the regulation of AhR/NF-κB/NLRP3 inflammasome pathway by VD3.

## Introduction

Periodontitis is a bacterium-induced chronic immunoinflammatory disease that leads to loss of gingival tissue and bone support of the dentition.^1^ *Porphyromonas gingivalis* (*P. gingivalis*) has been reported to be important in periodontitis and it can release virulence factors, such as lipopolysaccharide, inducing host immune response and periodontal tissue damage.^2^ After being exposed to lipopolysaccharide from *P. gingivalis*, gingival epithelial cells produced a variety of inflammatory mediators, including interleukin-1β (IL-1β) and interleukin-6 (IL-6), exerting various immunomodulatory functions in periodontal tissues.^3^^,^^4^ Thus, regulating the inflammatory response in gingival epithelia may become a potential strategy for periodontitis treatment.

Recently, vitamin D_3_ has emerged as a crucial regulator of the immune system, and it has immunomodulatory properties in different inflammatory diseases, including inflammatory bowel disease and oral lichen planus.^5^^,^^6^ Active form of vitamin D_3_, 1,25-dihydroxyvitamin D_3_ (VD3), is reported to alleviate excessive inflammatory response in many epithelial cells, including intestinal epithelial cells and keratinocytes.^6^^,^^7^ Although in clinical research, the beneficial effect of VD3 on periodontitis still remains to be confirmed;^8^^,^^9^ reduced alveolar bone loss has been observed in murine experimental periodontitis after supplementation with the stable form of VD3.^10^^,^^11^ These findings suggest that VD3 may have potential protective effect on periodontitis, and this effect might be linked to its immunomodulatory functions on the oral epithelium.

Aryl hydrocarbon receptor (AhR) is a ligand-activated transcription factor, and its immunoregulatory function has been highlighted recently.^12^ AhR activation has been shown to improve immune homeostasis in epithelial cells, and stimulation of oral commensal bacteria can enhance its activation in oral epithelial cells.^13^^,^^14^ NLR pyrin domain-containing 3 (NLRP3) is a pattern recognition receptor with a key role in host defense against pathogens.^15^ NLRP3 assembles a multi-protein complex (inflammasome), which consists of NLRP3, apoptosis-associated speck-like protein (ASC) and caspase-1.^16^ Current research on macrophages has shown that the NLRP3 inflammasome has a crosstalk with AhR and nuclear factor-κB (NF-κB), a pivotal regulator of inflammation-related gene transcription.^17^^,^^18^ Moreover, the activation of AhR signaling can be increased by VD3 in immune cells, including kidney epithelium-derived cells.^19^ These findings indicate that VD3 might regulate inflammatory response in periodontitis through modulating AhR/NF-κB/NLRP3 inflammasome signaling pathway.

This study aimed to explore the effect of VD3 treatment on periodontitis and AhR/NF-κB/NLRP3 inflammasome pathway in the gingival epithelium of C57BL/6 wild-type mice with experimental periodontitis induced by *P. gingivalis* inoculation.

## Methodology

### Animals and experimental groups

Thirty male C57BL/6 wild-type mice (6-wk-old, body weight 20-22 g) were obtained from Experimental Animal Laboratory, Guangxi Medical University (Nanning, China), and fed with standard laboratory chow and water *ad libitum*. After 1 wk acclimatization before the experiments, all mice were randomly assigned to normal control (N), *P. gingivalis* infection (P), or *P. gingivalis* infection with VD3 treatment (V) groups (10 mice in each group). The random division was done using the Statistical Package for the Social Sciences (SPSS) software (Version 20.0, SPSS Inc., Chicago, IL, USA), according to the instructions. All experiments were approved by the Institutional Animal Care and Use Committee of Guangxi Medical University (#20150303-4), and all protocols were in accordance with the ARRIVE (Animals in Research: Reporting in Vivo Experiments) guidelines.^20^

### Oral inoculation of *P. gingivalis*

A clinical strain ATCC 33277 of *P. gingivalis* (State Key Laboratory of Oral Diseases, Sichuan University, Chengdu, China) was cultured on blood agar with hemin/menadione (Sigma-Aldrich Co., St. Louis, MO, USA) under anaerobic conditions. At the age of 7 weeks, the mice in P and V groups were orally inoculated with 100 μl phosphate buffered saline (PBS) containing 2% carboxymethylcellulose and 10^9^ colony-forming units of live bacteria, 3 times at 2-day intervals within 5 days.^10^^,^^11^ The mice in N group received 100 μl PBS with 2% carboxymethylcellulose.^10^^,^^11^

### Treatment with VD3

The mice in V group were intraperitoneally injected with VD3 (Sigma-Aldrich Co., St. Louis, MO, USA) every other day from 11 wk of age, and they were injected the last time 1 day before sacrifice, at wk 19. VD3 was dissolved in sterile corn oil (VD3 dose: 2.5 μg/kg body weight), and sterile corn oil was used as vehicle (the mice in N and P groups were given only corn oil). All thirty mice were sacrificed by overdose of diethyl ether inhalation at the end of the experiment.

### Quantification of bone loss

Upon sacrifice, mouse mandibular jaws were dissected and alveolar bone loss of the first and second molars was examined using scanning electron microscopy. The area bordered by the cementoenamel junction, the alveolar bone crest, and the mesial and distal line angles on the lingual sides of the first and second molars of mandibular jaws was regarded as bone loss and quantified.^11^ The blind assessments by 2 technicians were repeated 3 times. The averaged data from both mandibles were calculated for representing the bone loss *per* animal.^11^

### Western blot analysis

Both mandibular gingival epithelial tissues of each mouse were separated using Dispase Ι (Sigma-Aldrich Co., St. Louis, MO, USA).^11^ Western blot analyses were performed according to the instructions. The primary antibodies were mouse monoclonal anti-glyceraldehyde 3-phosphate dehydrogenase (anti-GAPDH) (1:500), anti-vitamin D receptor (anti-VDR) (1:200), anti-AhR (1:500), anti-cytochrome P450 1A1 (anti-CYP1A1) (1:300), anti-NF-κB p65 (anti-p65) (1:500), anti-phospho-NF-κB p65 (anti-p-p65) (1:500), anti-ASC (1:500), anti-caspase-1 (1:500), anti-IL-1β (1:500), anti-IL-6 (1:500), and rabbit polyclonal anti-NLRP3 (1:500). The secondary antibody was horseradish peroxidase-conjugated anti-mouse (1:2000) or anti-rabbit (1:3000). The immunoreactive bands were detected using enhanced chemiluminescence. Except the rabbit polyclonal primary antibody from Abcam (Cambridge, MA, USA), all antibodies were from Santa Cruz Biotechnology (Santa Cruz, CA, USA).

### Immunohistochemical analysis

Mouse maxillae were fixed in 10% formalin, decalcified in 10% EDTA, embedded in paraffin, and cut into serial sections (5 μm) for immunohistochemical staining. The primary antibodies anti-VDR (1:100), anti-AhR (1:200), anti-CYP1A1 (1:200), anti-p-p65 (1:200), anti-NLRP3 (1:200), anti-ASC (1:200), anti-caspase-1 (1:200), anti-IL-1β (1:100), and anti-IL-6 (1:100), and the secondary antibodies (1:1000) were incubated with the section. All antibodies were purchased from Santa Cruz Biotechnology (Santa Cruz, CA, USA), except the anti-NLRP3 antibody (Abcam, Cambridge, MA, USA). Mean optical density of the staining was calculated using Image-Pro Plus software (Version 6.0, Media Cybernetics, Silver Spring, MD, USA), and the measurements obtained from both sides were averaged to represent each sample.^11^

### Statistical methods

Data were shown as the mean ± standard deviation. Statistical analysis of differences among groups was determined using one-way analysis of variance (ANOVA) testing, followed by Student-Newman-Keuls-*q* multiple comparisons. Prior to application of one-way ANOVA, normal distribution of the data was verified by values of Skewness and Kurtosis detection, and homogeneity of variances was tested by Levene's Test. A *P*-value<0.05 was accepted as significant.

## Results

### VD3 reduces alveolar bone loss in experimental periodontitis

**Figure 1:**
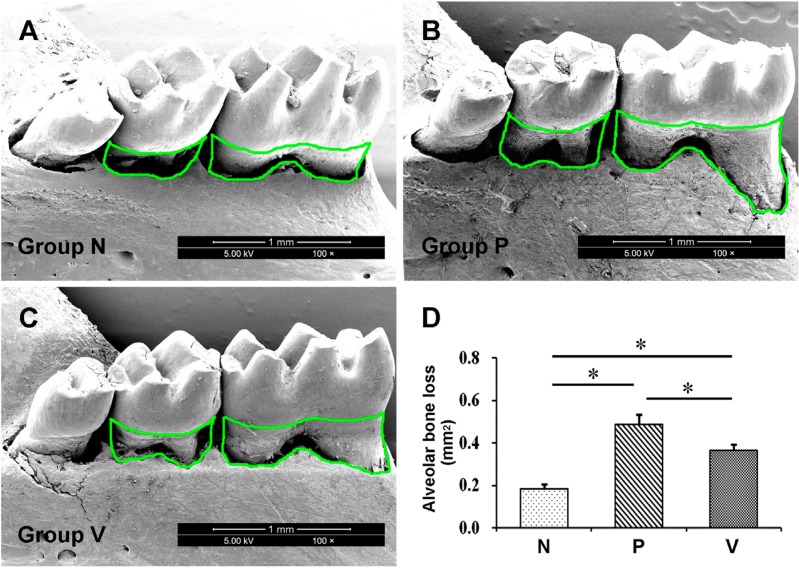
Alveolar bone loss of the lingual side of mouse mandibular first and second molars was measured using scanning electron microscopy. Bone loss was represented by the area bordered by the green lines. Treatment with 1,25-dihydroxyvitamin D3 decreased the bone loss in periodontitis mice. Values are means ± SD (n=10). *P<0.05. N, normal control; P, Porphyromonas gingivalis infection; V, *Porphyromonas gingivalis* infection with 1,25-dihydroxyvitamin D_3_ treatment

Quantitative analysis of alveolar bone loss revealed that both *P. gingivalis*-infected groups (V and P groups) had more bone loss than the normal control group. However, V group showed obviously decreased bone loss after VD3 administration for 8 wks when compared to P group (Figure 1). This result indicates the attenuated bone loss after VD3 treatment.

### VD3 increases VDR expression in gingival epithelia

**Figure 2:**
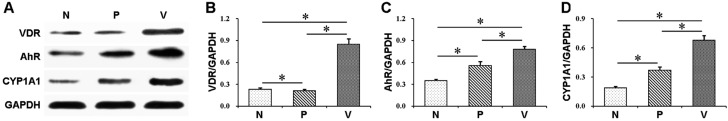
Protein expression of VDR, AhR and CYP1A1 in the mouse gingival epithelium was examined using western blot analysis. Treatment with 1,25-dihydroxyvitamin D3 enhanced VDR expression and increased AhR and CYP1A1 production. Values are means ± SD (n=3). *P<0.05. N, normal control; P, *Porphyromonas gingivalis* infection; V, *Porphyromonas gingivalis* infection with 1,25-dihydroxyvitamin D_3_ treatment

**Figure 3:**
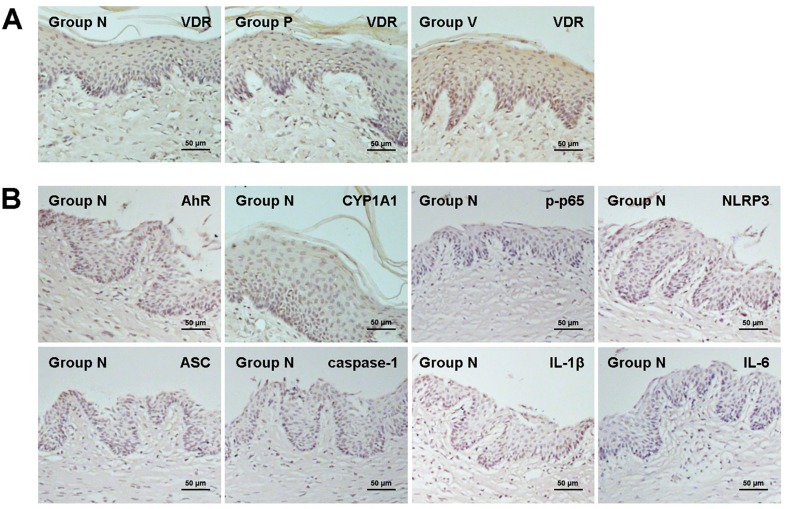
Gingival epithelial VDR expression in each group, and AhR, CYP1A1, p-p65, NLRP3, ASC, caspase-1, IL-1β and IL-6 expression in normal control group was shown in immunohistochemical images. Treatment with 1,25-dihydroxyvitamin D3 enhanced VDR expression, while the VDR expression exhibited no significant difference between normal control and untreated periodontitis mice. N, normal control; P, *Porphyromonas gingivalis* infection; V, *Porphyromonas gingivalis* infection with 1,25-dihydroxyvitamin D_3_ treatment

As shown by western blot analyses and immunohistochemical staining, VDR protein levels in the gingival epithelium were markedly increased in V group compared with other two groups (N and P groups). Moreover, no significant difference in VDR expression was observed between N and P groups (Figure 2, Figure 3, and Table 1).

**Table 1:** Mean optical density of the mouse gingival epithelium

	VDR	AhR	CYP1A1	p-p65	NLRP3	ASC	caspase-1	IL-1β	IL-6
N	0.095±0.004	0.091±0.005	0.075±0.005	0.095±0.005	0.081±0.005	0.102±0.003	0.077±0.006	0.087±0.005	0.099±0.004
P	0.092±0.003	0.146±0.004	0.103±0.005	0.187±0.006	0.154±0.005	0.179±0.005	0.145±0.007	0.194±0.007	0.218±0.008
V	0.148±0.003	0.173±0.004	0.142±0.008	0.151±0.004	0.112±0.006	0.134±0.004	0.110±0.005	0.138±0.006	0.173±0.004

"Note: Protein expression of VDR, AhR, CYP1A1, p-p65, NLRP3, ASC, caspase-1, IL-1β and IL-6 in the gingival epithelium of the mouse model was examined using immunohistochemical staining. Staining intensity was quantified, and shown as mean optical density. After 1,25-dihydroxyvitamin D3 treatment, the expression of VDR, AhR and CYP1A1 was enhanced in mice with periodontitis, while the expression of p-p65, NLRP3, ASC, caspase-1, IL-1β and IL-6 was inhibited. Data are presented as the mean ± SD (n = 10). VDR: P<0.05 for P vs. V mice, and N vs. V mice. AhR, CYP1A1, p-p65, NLRP3, ASC, caspase-1, IL-1β and IL-6: P<0.05 for N vs. P mice, P vs. V mice, and N vs. V mice. N, normal control; P, *Porphyromonas gingivalis* infection; V, *Porphyromonas gingivalis* infection with 1,25-dihydroxyvitamin D3 treatment.

### VD3 regulates AhR/NF-κB/NLRP3 inflammasome pathway in gingival epithelia

The findings of western blot analysis and immunohistochemical staining of AhR signaling are demonstrated in Figures 2, 3, 5 and Table 1. VD3 treatment increased *P. gingivalis* infection-induced AhR upregulation in gingival epithelia. Differences in AhR expression were significantly found between P mice and N mice, and between V mice and P mice. Similarly, the expression of AhR downstream CYP1A1 was greater in P mice than in N mice, and greater in V mice than in P mice. These data suggest the positive crosstalk of VD3 with AhR activation.

**Figure 4:**
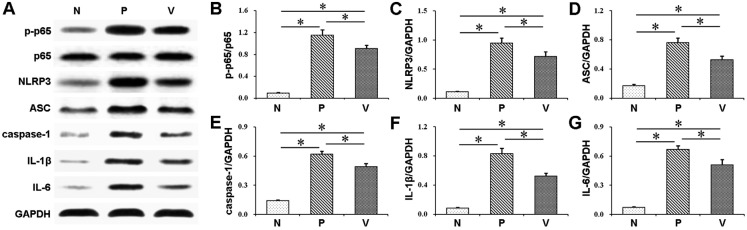
Phosphorylation of NF-κB p65 and protein expression of NLRP3, ASC, caspase-1, IL-1β and IL-6 in the mouse gingival epithelium were examined using western blot analysis. Treatment with 1,25-dihydroxyvitamin D3 inhibited NF-κB p65 phosphorylation and decreased NLRP3, ASC, caspase-1, IL-1β and IL-6 expression. Values are means ± SD (n=3). *P<0.05. N, normal control; P, Porphyromonas gingivalis infection; V, Porphyromonas gingivalis infection with 1,25-dihydroxyvitamin D_3_ treatment

**Figure 5:**
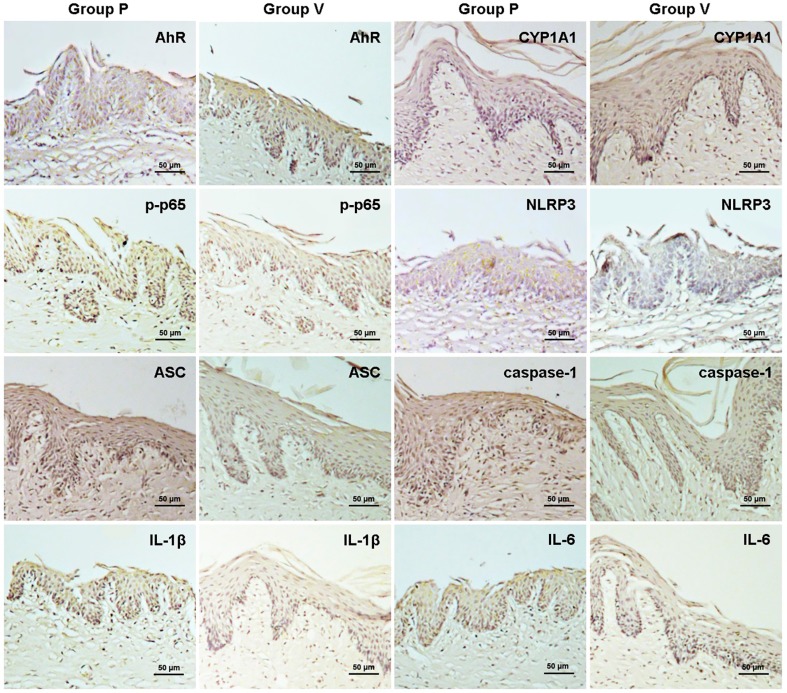
Protein expression of AhR, CYP1A1, p-p65, NLRP3, ASC, caspase-1, IL-1β and IL-6 in the mouse gingival epithelium was detected using immunohistochemical staining. After 1,25-dihydroxyvitamin D3 administration, the gingival epithelial expression of AhR and CYP1A1 was increased in mice with periodontitis, whereas the expression of p-p65, NLRP3, ASC, caspase-1, IL-1β and IL-6 was decreased. N, normal control; P, *Porphyromonas gingivalis* infection; V, *Porphyromonas gingivalis* infection with 1,25-dihydroxyvitamin D_3_ treatment

The results in Figures 3, 4, 5 and Table 1 demonstrate that untreated mice with periodontitis exhibited obviously elevated NF-κB phosphorylation levels in the gingival epithelium, compared to the normal controls (P *vs.* N). Additionally, the periodontitis mice with VD3 treatment showed reduced NF-κB phosphorylation levels comparing to their untreated unhealthy counterparts (V *vs.* P), which indicate suppression of NF-κB phosphorylation by VD3.

To evaluate the activation of NLRP3 inflammasome, NLRP3, ASC and caspase-1 expression in gingival epithelia were measured using western blot analysis and immunohistochemical staining. Western blot analyses showed that the expression of NLRP3, ASC and caspase-1 in gingival epithelia was enhanced in P group compared to N group, while decreased in V group compared to P group. The result of immunohistochemical staining was consistent with that of western blot analyses (Figures 3, 4, 5 and Table 1).

### VD3 inhibits IL-1β and IL-6 production in gingival epithelia

Western blot analyses showed amplified expression of IL-1β and IL-6 in P group compared with N group. Furthermore, attenuated expression of these cytokines was found in V group compared with P group (Figure 4). Similarly, immunohistochemical staining revealed that the expression of both cytokines was greater in mice with periodontitis than in their normal counterparts (P *vs.* N, and V *vs.* N), while the expression was reduced after VD3 treatment (V *vs.* P) (Figure 3, Figure 5 and Table 1).

## Discussion

Recently, VD3 has been considered an important regulator of immune response in different inflammatory diseases, such as type 1 diabetes and inflammatory bowel disease.^5^^,^^21^ In clinical research, vitamin D supplementation was reported to decrease the incidence of upper respiratory infection in patients with inflammatory bowel disease.^22^ Studies on animals with experimental periodontitis also showed that supplementation with the stable form of VD3, 25-hydroxyvitamin D_3_ could reduce alveolar bone loss, an important index of periodontitis.^10^^,^^11^ Here, we observed that alveolar bone loss was decreased upon VD3 treatment, indicating the inhibitory effect of VD3 supplementation on periodontitis.

Like other vitamins, vitamin D has its therapeutic dose range, and the recommended vitamin D doses may range between 400 and 2000 IU/day.^23^ Consuming too much vitamin D can result in toxicity, such as hypercalcemia, leading to cardiovascular injury and calcium deposition in soft tissues.^24^ However, some researchers showed no observed-adverse-effect of the vitamin D3 dose of 10000 IU/day, indicating the safety of this vitamin supplementation.^25^ In clinical practice, there are several factors affecting the recommended dose, and the wise choice of the recommendation to humans should depend on individual health outcome concerns, body weight, dietary, etc.^23^ Thus, the VD3 dose we chose in this work is just to provide a reference for the future study.

An increasing amount of literature has highlighted the immunoregulatory function of gingival epithelium in periodontitis, for it can produce various inflammation-related proteins.^26^^,^^27^ In this study, we showed amplified VDR expression in gingival epithelia, accompanied with decreased bone loss after VD3 administration, suggesting that the attenuated periodontal damage in periodontitis may be due to the interaction between VDR and other inflammation-related proteins in gingival epithelia. VDR is a key protein in VD3 signaling, and widely exists in epithelial cells, including intestinal and corneal epithelial cells.^28^^,^^29^ After binding to VD3, VDR becomes heterodimerized with the retinoid X receptor and regulates the synthesis of inflammatory proteins.^30^

In addition, we found that the expression of AhR and its downstream CYP1A1 was upregulated in gingival epithelia in periodontitis mice compared with their normal controls. Upon VD3 treatment, the expression was further enhanced. These data suggest that VD3 may inhibit periodontitis through increasing the AhR signaling activation. AhR is newly reported to improve immune homeostasis in diseases related to excessive proinflammatory status, such as Crohn's disease.^31^ Upon binding to its ligand, AhR activates the transcription of target genes including CYP1A1 and other downstream inflammatory proteins.^32^ AhR signaling can be activated by some products of bacteria, including lipopolysaccharide in immune cells, and its enhanced activation is found to correlate with the alleviation of inflammatory response.^12^^,^^33^ In monocytic cells, VD3 can increase the activation of AhR signaling, possibly through binding to the promoter sequences required for the activation of this signaling.^19^

Furthermore, we found that the phosphorylation of NF-κB p65 and the expression of NLRP3, ASC and caspase-1 were increased in the gingival epithelium in periodontitis, while they were decreased upon VD3 treatment. These observations indicate that the periodontitis attenuation by VD3 may involve the repression of NF-κB and NLRP3 inflammasome activation by AhR signaling. NF-κB is crucial in the major signaling networks for inflammatory response modulation.^34^ As an important member of NF-κB family, NF-κB p65 can be significantly activated by lipopolysaccharide, the major virulence factor of *P. gingivalis*, and its phosphorylation was greatly associated with periodontal damage.^35^ Upon stimulation by lipopolysaccharide, NF-κB becomes phosphorylated, and binds to the NF-κB binding sites in the NLRP3 promoter region, resulting in the NLRP3 inflammasome activation in immune cells.^36^ In macrophages, the activation of AhR signaling blocks NF-κB binding sites and masks NF-κB transcription activity, subsequently suppressing NLRP3 inflammasome activation.^17^

In our experiments, the IL-1β and IL-6 expression in gingival epithelia was elevated in all periodontitis mice compared with their normal controls. Moreover, reduced expression of these cytokines occurred upon VD3 injection. These findings implicate that the alleviation of experimental periodontitis by VD3 may be partly due to the inhibition of IL-1β and IL-6 production, which may result from the change in AhR/NF-κB/NLRP3 inflammasome pathway. Previous research has shown the important role of IL-1β in the host defense against bacterial infections and the pathogenesis of periodontitis.^37^ It can strongly promote the IL-6 production, a potent stimulator of alveolar bone resorption in this periodontal disease.^38^ IL-6 upregulates the expression of RANKL (osteoclast differentiation factor), and promotes osteoclastogenesis.^39^ Lipopolysaccharide can trigger the phosphorylation of NF-κB, and subsequently leads to the activation of NLRP3 inflammasome pathway, which processes pro-IL-1β into its mature form IL-1β.^17^^,^^40^ In immune cells such as macrophages, through the interaction with NF-κB, activated AhR signaling can inhibit NLRP3 inflammasome formation and subsequent secretion of IL-1β, and other proinflammatory cytokines, improves inflammatory response.^17^

In clinical research, the VD3 effect on periodontal disease still remains controversial. Several studies on adults showed that lower serum 25-hydroxyvitamin D_3_ levels were significantly associated with periodontitis.^41^^,^^42^ However, some investigators demonstrated that serum vitamin D levels or vitamin D supplementation did not seem to be related to periodontal status.^8^^,^^43^ These different findings may partly be attributed to wrong study designs, short follow-up duration or cross-sectional design, poorly paired case-controls, and incorrect statistical modeling, etc. Thus, to clinically confirm the VD3 effect on periodontal health, long follow-up and more well-designed randomized trials are still needed.

Compared with human clinical studies, researches on animal models have less uncontrollable variables, so a variety of animal models have been used for investigating periodontal diseases. Ligature models and oral gavage models are two important models for experimental periodontitis study. Ligature placement causes massive local bacterium accumulation and sulcular epithelial microulceration, facilitating bacterium invasion into deeper periodontal tissues.^44^ This model can be used to mimic the periodontal destruction induced by indigenous periodontal pathogens-stimulated host response in human periodontitis,^44^ but the ligatures may be loose/lost, so they need to be checked or even be replaced during the study. Oral gavage of bacteria is a convenient method to establish periodontitis models in some mouse strains. It can introduce human strains of bacteria, such as *P. gingivalis*, into mouse periodontal tissues, and be used for studying the subsequent impact on the periodontium.^44^ However, different mice strains have different susceptibility to experimental periodontal disease. It has been reported that C57BL/6 mice are more susceptible, and the difference in susceptibility is associated with mouse genetic variances influencing immune response.^45^ This finding suggests the utilization of more animals with different genetic background to further elucidate the mechanisms of pathogenesis and treatment in periodontitis.

*P. gingivalis* is not natural in mouse oral microbial community, but its oral inoculation has been used to induce experimental periodontitis in mice. It was reported that the immune defense status in mice had some similarity to that in human, allowing the research on immune response to human periopathogens using mouse models.^46^ Certain mouse strains, such as C57BL/6 mice, are more susceptible to *P. gingivalis*, so it is convenient to use them to establish periodontitis models in a short time and to detect the effect of periodontal treatment. Previous reports have shown the potential association between *P. gingivalis* and periodontitis.^47^^,^^48^ *P. gingivalis* introduction increased the total oral microbial load and changed its composition in rabbit and non-human primate models of periodontitis.^47^^,^^48^ Moreover, immunization using an antigen from *P. gingivalis* reduced the oral microbial load and the alveolar bone loss in non-human primates.^48^ However, recent research project showed that the onset and progression of periodontitis could be modified by various host response genes.^49^ Specific bacteria and their virulence factors cannot guarantee periodontitis progression, though they are necessary for its development.^50^^,^^51^ Early host-inflammatory and immune response may be more important in the determination of periodontitis development.^50^^,^^51^ These reports highlighted the central role of gene background and immune response in the periodontitis pathogenesis. Thus, in the study on periodontitis, especially on its inflammatory regulation, we should consider the genetic differences between different animal species, and between experimental animals and humans.

This study showed the VD3 effect on experimental periodontitis in a mouse model, but there were some limitations. First, the mouse strain we used was more susceptible to human oral bacterium *P. gingivalis* and tended to exhibit severe periodontitis, compared with some other mouse strains and animal species. Second, we did not compare the *in vivo* VD3 concentrations before and after VD3 treatment to show the its supplementation levels. Third, we did not examine some inflammatory markers closely related to periodontitis, such as periodontal inflammatory infiltrate in mice. To further elucidate the precise effect and mechanisms of VD3 on periodontitis, in future experiments we can choose different animals to induce periodontitis, such as different mouse strains with AhR knockdown or rat ligature models, to mimic the complex human host response to microbial challenge and treatment. Moreover, we can detect more parameters, as periodontal VD3 concentrations and inflammatory infiltrate in periodontal tissues, to confirm the VD3 status and periodontal inflammation.

## Conclusions

We observed that VD3 attenuated *P. gingivalis*-induced periodontitis in mice. Additionally, VD3 enhanced AhR activation and suppressed the activation of NF-κB and NLRP3 inflammasome in the gingival epithelium. These results suggest the inhibition of experimental periodontitis and alteration of AhR/NF-κB/NLRP3 inflammasome pathway by VD3. The protective VD3 effect on periodontitis may correlate with the regulation of AhR/NF-κB/NLRP3 inflammasome pathway.
